# Clinical outcomes of 188 patients implanted with Med-El stapes prostheses

**DOI:** 10.1007/s00405-025-09733-x

**Published:** 2025-10-15

**Authors:** Wendelin Wolfram, Paul Martin Zwittag, Lisa Niederwanger, Nina Rubicz, Georg Sprinzl, Astrid Magele, Dirk Beutner, Nicholas Bevis, Esther Schimanski, Susan Arndt, Christian Offergeld, Christoph Arnoldner, Dominik Riss, Piotr H. Skarżyński, Łukasz Plichta, Joachim Hornung, Lava Taha, Thomas Lenarz, Susan Busch, Franz Windisch, Benjamin Loader

**Affiliations:** 1 Department of Otorhinolaryngology, Klinikum Wels-Grieskirchen, 4600 Wels, Austria; 2 Department of Otorhinolaryngology, Head and Neck Surgery, Kepler University Hospital GmbH, 4020 Linz, Austria; 3 Medical Faculty, Johannes Kepler University Linz, Altenbergerstrasse 69, 4040 Linz, Austria; 4 Department of Otorhinolaryngology, Head & Neck Surgery, University Clinic St. Poelten, 3100 St. Pölten, Austria; 5 Department of Otolaryngology, University of Goettingen, 37075 Göttingen, Germany; 6 Zentrum Fuer Mittelohrchirurgie (Centre for Middle Ear Surgery), ENT Practice, 44536 Luenen, Germany; 7 Department of Otorhinolaryngology-Head and Neck Surgery, Faculty of Medicine, Medical Center-University of Freiburg, University of Freiburg, 79106 Freiburg, Germany; 8 Department of Otorhinolaryngology, Medical University of Vienna, 1090 Vienna, Austria; 9 Clinical Trials Department, Center of Hearing and Speech MEDINCUS, Kajetany, Poland; 10 Institute of Sensory Organs, Kajetany, Poland; 11 World Hearing Center of the Institute of Physiology and Pathology of Hearing, Kajetany, Poland; 12 Department of Teleaudiology and Screening, World Hearing Center, Institute of Physiology and Pathology of Hearing, 10 Mochnackiego Street, 02-042 Warsaw, Poland; 13 Department of Otorhinolaryngology, Head & Neck Surgery, University of Erlangen-Nuremberg, 91054 Erlangen, Germany; 14 Department of Otolaryngology, Hannover Medical School, 30625 Hannover, Germany; 15 Cluster of Excellence Hearing4all, Medical University Hannover, 30625 Hannover, Germany; 16 Department of Otorhinolaryngology, Head and Neck Surgery, Klinik Landstraße, Wiener Gesundheitsverbund, 1030 Vienna, Austria; 17 Sigmund Freud Private University, 1020 Vienna, Austria

**Keywords:** Stapedotomy, Stapedectomy, Otosclerosis, Hearing restoration, Passive middle ear implants, MAXIS Stapes Prosthesis, MLOOP Stapes Prosthesis, MZAM Stapes Prosthesis, MFIX Stapes Prosthesis

## Abstract

**Purpose:**

This multicentric, retrospective study aimed to analyze the safety and effectiveness of the mAXIS Stapes Prosthesis, mLOOP Stapes Prosthesis, mZAM Stapes Prosthesis, and mFIX Stapes Prosthesis.

**Methods:**

Patients underwent stapes surgery and implantation of a mAXIS Stapes Prosthesis, mLOOP Stapes Prosthesis, mZAM Stapes Prosthesis, or mFIX Stapes Prosthesis (MED-EL, Innsbruck, Austria). The clinical data was retrospectively analyzed. Follow-up examination included access to medical records (for adverse events) of the patients, ear microscopy and pure-tone audiometry to determine the post-operative pure tone average of the frequencies 0.5, 1, 2 and 3 kHz (PTA_4_). The post-operative PTA_4_ air bone gap (ABG) was used to evaluate the audiological outcome. A post-operative PTA_4_ ABG ≤ 20 dB was defined as successful rehabilitation. A post-operative minimum and maximum follow-up period was not defined.

**Results:**

189 patients were implanted with a MED-EL stapes prosthesis mainly as treatment of hearing loss caused by otosclerosis. 188 (186 adults, 2 children; 57 conductive hearing loss (CHL), 131 mixed hearing loss (MHL)) patients were examined for adverse events (AEs). 168 (166 adults, 2 children, 51 CHL, 117 MHL) patients underwent audiological examination. Audiology: 110 (65.5%) patients achieved a post-operative PTA_4_ ABG ≤ 10 dB. 154 (91.7%) patients achieved a post-operative PTA_4_ ABG ≤ 20 dB and therefore successful rehabilitation. Individual bone conduction (BC) PTA_4_ thresholds were stable in 159 (94.6%) patients. AEs: 12 (6.4%, adults only) patients had 13 AEs.

**Conclusion:**

Clinical data demonstrated satisfactory audiological results after implantation of the mAXIS Stapes Prosthesis, mLOOP Stapes Prosthesis, mZAM Stapes Prosthesis, and mFIX Stapes Prosthesis. The MED-EL stapes prostheses are safe and effective.

**Trial registration number:**

NCT05565339 (clinicaltrials.gov).

**Supplementary Information:**

The online version contains supplementary material available at 10.1007/s00405-025-09733-x.

## Introduction

At the 6th International Congress of Otology in London in 1889, Adam Politzer reported on the risks of meningitis and death after stapes surgery [1]. Leading otologists at the time denounced stapes surgery for otosclerosis as “useless and dangerous” [2]. Many important historical developments in stapes surgery have been made since then. In 1890, Samuel Sexton introduced an electric head lamp for use during ossicular surgery [3]. In 1922, Gunnar Holmgren adapted an operating microscope for otosclerosis surgery use [4]. The first widely-used audiometer, The Western Electric 2-A, was developed in 1923 by Harvey Fletcher and R.L. Wegel [5]. The first stapedectomy was performed in 1955 by Shea [6]. In 1961, Robinson designed a bucket-style incus receptacle, which was followed in 1962 by the bucket prosthesis designed by Shea [7]. The first Teflon wire piston was developed by Schuknecht in 1961 [7], with the first wire loop prosthesis introduced by House in 1962 [8]. The first piston stapes prosthesis made of titanium (Chicago titanium bucket handle) was designed in 1989 by the Goble brothers in England (Gyrus Medical) [9]. Patients with stapes footplate fixation, e.g., due to otosclerosis within the oval window niche, suffer from CHL. Patients may have an additional sensorineural hearing loss (SNHL) component, resulting in MHL [10]. The onset of otosclerosis is usually in the third to fourth decade of life [11]. The current state-of-the-art surgical procedures to treat otosclerotic hearing loss are stapedectomy and stapedotomy [12]. During these types of surgeries, the stapes footplate is either totally removed (stapedectomy) or punctured (stapedotomy). The stapes prosthesis is then inserted into the oval window (stapedectomy) or placed through the perforated footplate (stapedotomy). Subsequently, the oval window is sealed with soft tissue [13]. In the long term, stapedotomy results in better hearing gain at high frequencies and lower complication rates than stapedectomy [12]. Ossicular reconstruction using titanium prostheses is well established and state-of-the-art due to its excellent biocompatibility; nevertheless, materials such as Teflon, hydroxyapatite or nickel-titanium alloys are commonly used. Our study provides the first results on patients implanted with the new MED-EL titanium stapes prostheses: mAXIS, mLOOP, mZAM and mFIX (MED-EL, Innsbruck, Austria).

### Stapes prostheses designs

**Fig. 1:**
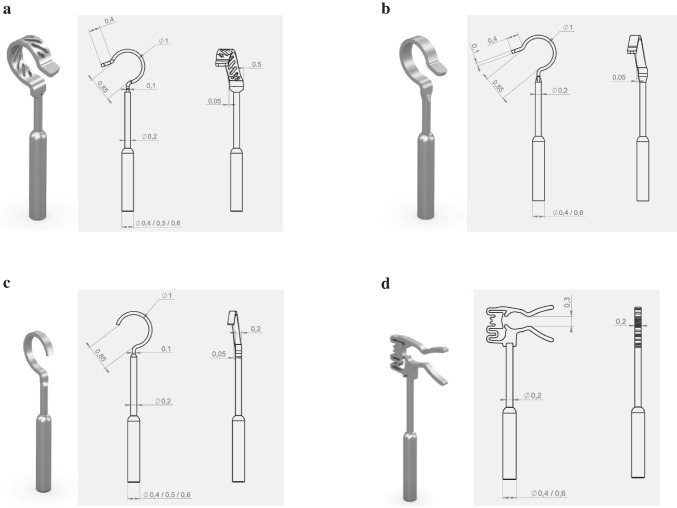
**Schematic drawing with technical data of the stapes prosthesis: a) mAXIS Stapes Prosthesis**: The wide loop, which reduces pressure on the incus, while perforations decrease the manual force needed for crimping. It has a bendable shaft and is available in 27 versions (different diameters (0.4/0.5/0.6 mm) and functional lengths (3.5, 3.75, 4.0, 4.25, 4.5, 4.75, 5.0, 5.25, 5.5 mm)). Attachment type: manual crimping. Weight: approx. 1.0–3.5 mg, depending on length. **b) mLOOP Stapes Prosthesis:** The prosthesis is available in lengths of up to 10 mm and can therefore be attached to both the incus and the malleus. The mLOOP also offers intra-operative flexibility with its offset band loop and bendable shaft as well as smooth transition between the piston and shaft. It is available in 28 versions (different diameters (0.4/0.6 mm) and functional lengths (3.5, 3.75, 4.0, 4.25, 4.5, 4.75, 5.0, 5.25, 5.5, 6.0, 7.0, 8.0, 9.0, 10.0 mm)). Attachment type: manual crimping. Weight: approx. 1.0–4.0 mg, depending on length. **c) mZAM Stapes Prosthesis:** The prosthesis has an offset band loop and a bendable shaft. It has an extremely slim design which allows for the best possible view when placing the implant. It is available in 27 versions (different diameters (0.4/0.5/0.6 mm) and functional lengths (3.5, 3.75, 4.0, 4.25, 4.5, 4.75, 5.0, 5.25, 5.5 mm)). Attachment type: manual crimping. Weight: approx. 1.0–3.5 mg, depending on length. **d) mFIX Stapes Prosthesis:** The mFIX Stapes Prosthesis provides crimp-free coupling with a clip, which means there is enough room between the implant and the posterior auditory canal wall. It is available in 18 versions (different diameters (0.4/0.6 mm) and functional lengths (3.5, 3.75, 4.0, 4.25, 4.5, 4.75, 5.0, 5.25, 5.5 mm)). Attachment type: clip. Weight: approx. 1.5–3.5 mg, depending on length

The titanium stapes prostheses mAXIS **(**Fig. 1a**),** mLOOP **(**Fig. 1b**)**, mZAM **(**Fig. 1c**)** and mFIX **(**Fig. 1d**)** have lightweight, rigid shafts and are MRI-compatible at 1.5 T (Tesla), 3.0 T and 7.0 T, according to the instructions disclosed by the manufacturer. Despite its rigidity, the shaft can be bent without special equipment. The prostheses consist of a coupling structure (incus end: loop or clip), a bendable shaft and an oval window attachment base (piston).

## Material and methods

### Ethical considerations

Our study was conducted in Germany, Austria and Poland in agreement with the Declaration of Helsinki and was approved by the relevant German, Austrian and Polish ethics committee(s) (Göttingen: 1/9/20; Hannover: 9456_BO_S_2020; Erlangen: 456_20 Bc; Lünen: 2020–829-b-S; Freiburg: 22–1142-retro; Linz: 1257/2022; Wels: 1257/2022; Gesundheitsverbund, Klinik Landstraße: EK_23_005_XX; AKH Vienna: 2296/2021; Sankt Pölten: GS1-EK-4/777–2022; Warsaw: Oświadczenie nr. 10/2023r.) as a post-market clinical follow-up study.

### Study design and number of patients

This multicenter, retrospective follow-up study included 189 patients (189 ears), with each patient serving as his or her own control. 188 of 189 patients were assessed for AEs (1 patient (patient 53) was excluded because of missing post-operative follow-up information). Audiological data was available for 168 of the 189 patients (21 of the 189 patients were excluded from the audiological analysis due to missing audiological data). For the analysis, patients implanted with the mAXIS Stapes Prosthesis, mLOOP Stapes Prosthesis, mZAM Stapes Prosthesis, or mFIX Stapes Prosthesis (MED-EL Innsbruck, Austria) (regardless of prosthesis length or piston diameter variant) until December 31, 2022, were included **(**Table 1**)**.

**Table 1:** Demographics

	**All stapes prostheses**	**mAXIS** **Stapes Prosthesis**	**mLOOP** **Stapes Prosthesis**	**mZAM** **Stapes Prosthesis**	**mFIX** **Stapes Prosthesis**
Number of patients included in the study	189	94	64	11	20
**AUDIOLOGY:**					
Total number of patients/children	168/2	88/0	50/1	11/1	19/0
Gender: female/male	115/53	61/27	38/12	6/5	10/9
Patient age at the time of implantation: mean ± SD (range) [years]	46.4 ± 12.9 (12–82)	46.0 ± 12.6 (21–77)	48.3 ± 12.0 (12–68)	43.8 ± 12.7 (14–60)	44.5 ± 16.5 (25–82)
Implanted ear: right/left	86/82	39/49	27/23	6/5	14/5
Type of HL before implantation: CHL/MHL	52/116	39/49	3/47	3/8	7/12
Follow-up time [days]: mean ± SD range median	64.6 ± 61.4 7–470 49.5	58.9 ± 48.1 7–253 43.5	62.7 ± 84.4 14–470 43.0	99.8 ± 32.5 69–180 90.0	75.5 ± 50.0 15–225 50.0
**ADVERSE EVENTS:**					
Total number of patients/children	188/2	93/0	64/1	11/1	20/0
Gender: female/male	127/61	65/28	46/18	6/5	10/10
Patient age at the time of implantation: mean ± SD (range) [years]	46.9 ± 12.9 (12–82)	46.3 ± 12.5 (21–77)	49.2 ± 12.2 (12–74)	43.8 ± 12.7 (14–60)	43.6 ± 16.6 (25–82)
Implanted ear: right/left	98/90	43/50	34/30	6/5	15/5
Type of HL before implantation: CHL/MHL	56/132	41/52	4/60	3/8	8/12
Follow-up time [days]: mean ± SD range median	246.4 ± 212.8 7–709 179.0	186.0 ± 180.3 7–666 92.0	276.7 ± 220.8 11–709 234.5	202.8 ± 232.0 68–701 104.0	454.6 ± 174.9 58–621 506.5

HL: hearing loss; CHL: conductive hearing loss; MHL: mixed hearing loss

### Follow-up time

Patients were evaluated pre- and post-operatively (1 pre- (BC thresholds) and 1 post-operative (BC- and air conduction (AC) thresholds) audiological measurement). A post-operative minimum and maximum follow-up period was not specified. This resulted in different follow-up times for audiological and AE analysis, depending on the study center. The mean post-operative follow-up time for all patients was calculated.

Post-operative PTA_4_ was calculated as a four-frequency mean of 0.5, 1, 2, and 3 kHz (PTA_4_), according to the American Academy of Otolaryngology-Head and Neck Surgery [14]. If there was no 3 kHz data available, 4 kHz data was used instead, without extrapolating the data.

### Audiometric methods

#### PTA_4_ ABG

A post-operative PTA_4_ ABG of ≤ 20 dB was defined as successful ABG closure [15].

#### BC PTA_4_

Pre- and post-operative BC PTA_4_ were calculated to determine the safety of the implantation.

### Adverse events (AE)

All surgical-, procedure- and device-related AEs in the operated ear that occurred intra- and post-operatively were reported.

### General Information

PTA_4_ ABG and AEs were analyzed descriptively. BC PTA_4_ was calculated inferentially. Graphs were created with GraphPad Prism 7.04 (GraphPad Software, Inc.).

## Results

### Demographics (N = 188)

The 188 patients (127 female, 61 male), including 2 children (patient 162,145), were treated in 11 clinics: 5 in Germany (Göttingen, Hannover, Erlangen, Lünen, Freiburg), 5 in Austria (Linz, Wels, Gesundheitsverbund Vienna, AKH Vienna, Sankt Pölten), and 1 in Poland (Kajetany). The mean age was 46.9 ± 12.9 years (range: 12–82 years) at the time of implantation. 98 (52.1%) patients were implanted in the right ear and 90 (47.9%) in the left ear. 57 (30.3%) patients suffered from CHL, and 131 (69.7%) from MHL (Online Resource: ESM 1: Overview of patients). For the separate analysis per prosthesis please see Table 1.

### Audiometric results (N = 168)

#### PTA_4_ ABG

The mean post-operative follow-up time was 64.6 ± 61.4 days (range: 7–470 days; median: 49.5 days). The mean post-operative PTA_4_ ABG was 9.1 ± 6.9 dB (range: −13.8–31.3 dB; median: 8.8 dB). 110 (65.5%) of the 168 patients had a PTA_4_ ABG of ≤ 10 dB (95% confidence interval (CI) 58.1–72.9%). 154 (91.7%) patients had a PTA_4_ ABG of ≤ 20 dB (95% confidence interval (CI) 84.3–99.1%). The mean post-operative PTA_4_ ABG of the remaining 14 (8.3%) patients that had a PTA_4_ ABG of > 20 dB was 25.0 ± 3.0 dB (range: 21.3–31.3 dB; median: 24.3 dB).

#### BC PTA_4_ thresholds

**Fig. 2:**
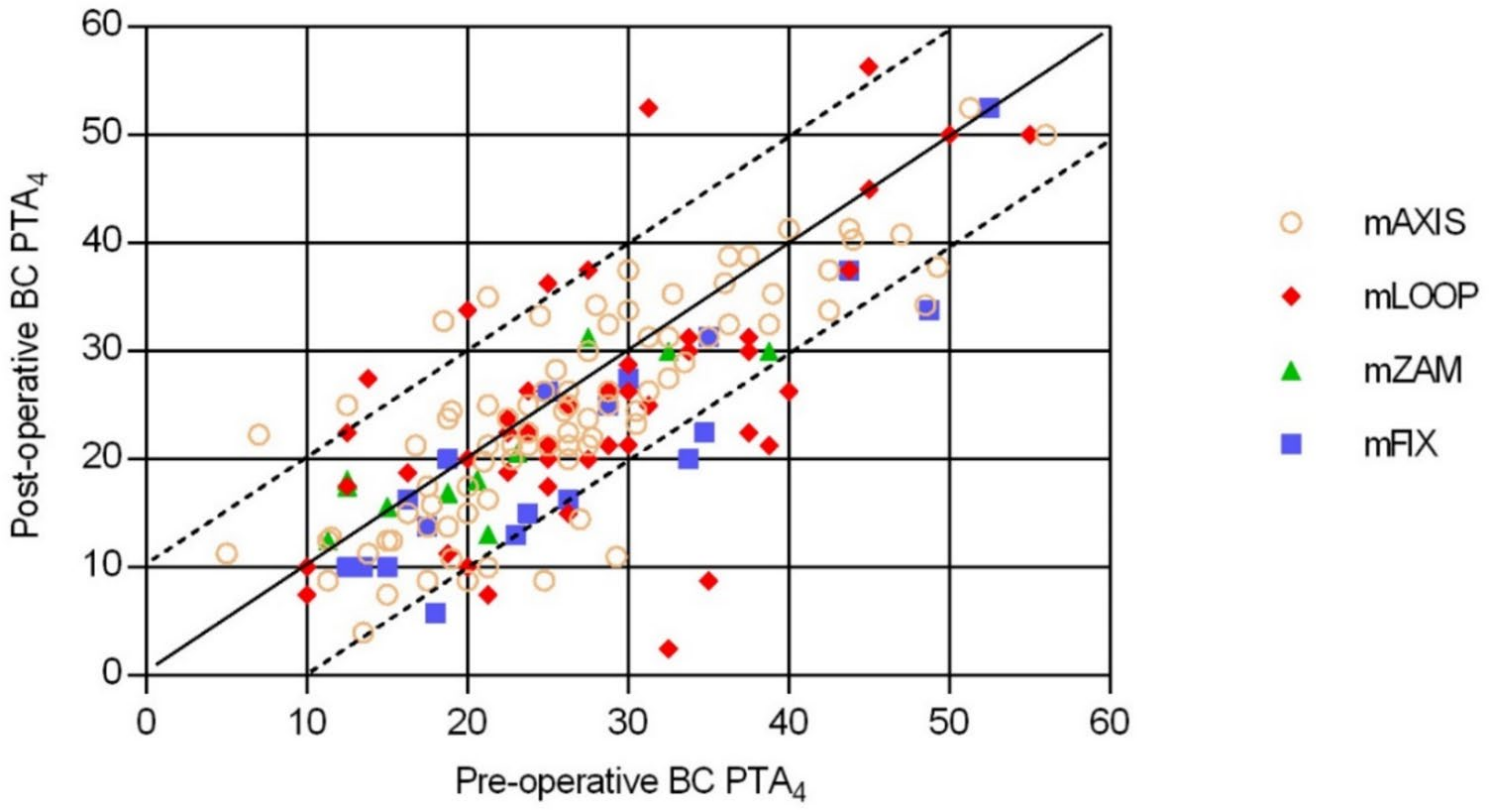
**Pre- and post-operative BC PTA**_**4**_** values of the mAXIS, mLOOP, mZAM and mFIX Stapes Prostheses:** Upper dashed line: above this line are patients with a post-operative BC PTA_4_ deterioration of > 10 dB HL. Below the upper dashed line are patients with a post-operative BC PTA_4_ deterioration of ≤ 10 dB HL. Straight line: patients with stable (unchanged) pre- and post-operative BC PTA_4_ values. Lower dashed line: above the line are patients with a post-operative BC PTA_4_ improvement of < 10 dB HL. Below the lower dashed line are patients with a post-operative BC PTA_4_ improvement of > 10 dB HL

The individual BC PTA_4_ thresholds were stable in 159 (94.6%) patients and within the test–retest fluctuation of ± 5 dB HL. 9 patients (patients 5, 12, 37, 38, 100, 108, 141, 142, 148) had a BC PTA_4_ deterioration of > 10 dB HL (range: 11.3–21.3 dB HL) after implantation (Fig. 2).

The mean BC PTA_4_ threshold of all 168 patients was pre-operative 26.6 ± 10.3 dB HL (range: 5.0–56.0 dB HL; median: 25.0 dB HL) and post-operative 23.9 ± 10.8 dB HL (range: 2.5–56.3 dB HL; median: 22.5 dB HL).

The mean post-operative PTA_4_ improvement per frequency was 2.0 dB HL at 0.5 kHz, 2.8 dB HL at 1 kHz, 5.9 dB HL at 2 kHz and 1.1 dB HL at 3 kHz; there was a deterioration of 1.4 dB HL at 4 kHz.

### Separate analysis per prosthesis

**Fig. 3:**
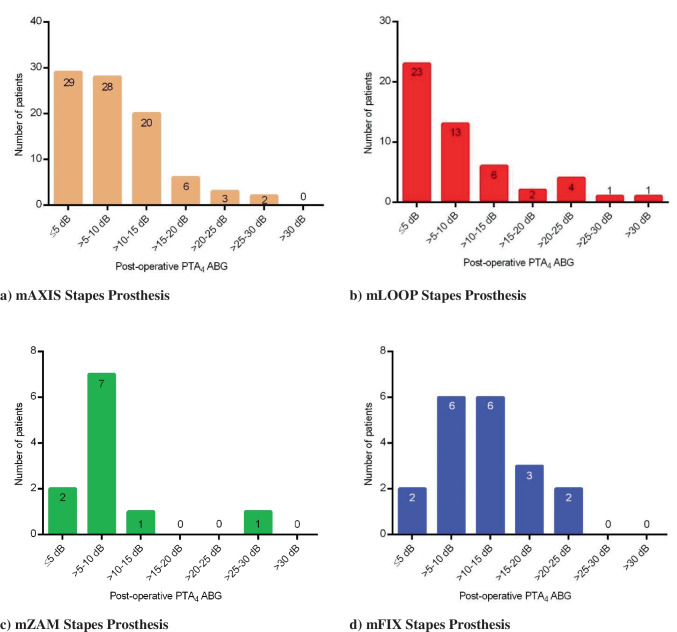
**a-d** Number of patients with the post-operative PTA_4_ ABG results, pictured in 5 dB intervals

- Please see Table 2 and Fig. 3a-d.

**Table 2:** Separate analysis per prosthesis

Type of stapes prosthesis	Follow-up time [days]			PTA_4_ ABG of ≤ 10 dB	PTA_4_ ABG of ≤ 20 dB	Mean post-op PTA_4_ ABG [dB]	Stable Δ BC PTA_4_ thresholds	Pre-op mean BC PTA_4_ [dB HL]	Post-op mean BC PTA_4_ [dB HL]
	mean ± SD	range	median						
**mAXIS** **(N = 88)**	58.9 ± 48.1	7–253	43.5	64.8% 57/88	94.3% 83/88	8.8 ± 6.0 range: −0.3–30.0	95.5% 84/88*	26.6 ± 10.3 range: 5.0–56.0	24.6 ± 10.2 range: 4.0–52.5
**mLOOP** **(N = 50)**	62.7 ± 84.4	14–470	43.0	72.0% 36/50	88.0% 44/50	8.3 ± 8.4 range: −13.8–31.3	90.0% 45/50**	27.7 ± 10.0 range: 10.0–55.0	24.2 ± 12.1 range: 2.5–56.3
**mZAM** **(N = 11)**	99.7 ± 31.8	68–180	90.0	81.8% 9/11	90.9% 10/11	9.0 ± 6.9 range: 1.3–28.1	100% 11/11	21.3 ± 8.8 range: 11.3–38.8	20.3 ± 6.9 range: 12.5–31.3
**mFIX** **(N = 19)**	75.5 ± 50.0	15–225	50.0	42.1% 8/19	89.5% 17/19	12.6 ± 5.9 range: 1.3–22.5	100% 19/19	27.2 ± 11.8 range: 12.5–52.5	21.4 ± 11.6 range: 5.8–52.5

^*^4 patients had a PTA_4_ BC deterioration of > 10 dB HL: patient 5 = 14.3 dB HL, patient 12 = 15.3 dB HL, patient 37 = 12.5 dB HL, patient 38 = 13.8 dB HL

^**^5 patients had a PTA_4_ BC deterioration of > 10 dB HL: patient 100 = 13.8 dB HL, patient 108 = 11.3 dB HL, patient 141 = 13.8 dB HL, patient 142 = 11.3 dB HL, patient 148 = 21.3 dB HL

Pre-op: pre-operative; post-op: post-operative; stable Δ BC PTA_4_: post-op minus pre-op BC PTA_4_ thresholds within ± 5 dB HL

### Adverse events (AEs) (N = 188)

The mean post-operative follow-up time was 246.4 ± 212.8 days (range: 7–709 days; median: 179.0 days). The mean post-operative occurrence time of the 13 AEs was 31.3 ± 26.1 days (range: 1–90 days) (for more details, please refer to (Table 3)). It was reported that 12 (6.4%, adults only) patients had 13 AEs (9 were resolved, 2 were not resolved, the status of 2 AEs was not available).

**Table 3:** Adverse events (N = 188)

Patient ID	Stapes prosthesis	Device related?	Occurrence time after surgery [days]	Event description	Treatment	Outcome
**163**	**mZAM**	**Unknown**	**1**	**Vertigo**	**Not known**	**Resolved**
29	mAXIS	No	17	Sensation of foreign body in the ear canal	Suction in the ear canal	Resolved
30	mAXIS	No	18	Sensation of foreign body in the ear canal	Cleaning and treatment with an antibiotic	Resolved
97	mLOOP	No	90	Cracking noise during jaw movement while lying down	Not known	NA*
99	mLOOP	No	10	Pressure, hearing reduction and autophony	Keeping the ear dry	Resolved
**100**	**mLOOP**	**No**	**15**	**Hearing loss: BC deterioration**	**Treatment with Urbason, intravenously**	**Resolved**
**101**	**mLOOP**	**Unknown**	NA*	**Subjective feeling of hearing deterioration**	**Unchanged pure tone audiogram; no conductive hearing loss**	NA*
34	mAXIS	No	24	Mild pain; fluid from the ear canal	Locally medication	Resolved
35	mAXIS	No	28	Fungal hyphae in the radical cavity	Cleaning and treatment with an antimycotic	Resolved
		No	70	Chronic recurrent otorrhea and local inflammation	Clearing, medication, follow-up appointment, revision surgery	Not resolved
102	mLOOP	No	21	Meniere's disease attack caused ipsilateral hearing deterioration (subjective impression)—started 4 days before appointment, tinnitus, vertigo. Patient has been suffering from Meniere’s disease for 17 years	Treatment with a glucocorticoid	Resolved
103**	mLOOP	No	50	Strong pain in the ipsilateral ear, relation to the ongoing Covid 19 infection is assumed	Not available	Resolved
140**	mLOOP	No	31	One month after surgery: tick bite, dizziness, hearing loss	Treatment with cortisone	Not resolved
**Mean post-operative occurrence time [days]**			**31.3 ± 26.1** **(range: 1–90)**			

^*^NA = Not available

^**^N = 2 (patient 103, 140) patients were followed-up for AEs only, and not for the audiology

## Discussion

### Reasons for implantation (N = 168)

In our study, 91.7% (N = 154) of the 168 patients were implanted with a stapes prosthesis due to otosclerosis. The remaining 8.3% were implanted because of revision of the prior prosthesis (3.6%; N = 6), dislocation of the prior prosthesis and incus necrosis (N = 1), and other factors (N = 7).

Other indications for stapes prosthesis implantation include osteogenesis imperfecta. A study by Skarzynski et al. utilized stapedotomy to treat hearing loss caused by osteogenesis imperfecta. The mean ABG before surgery was 32.2 ± 8.88 dB; at ≤ 12 months post-operative this was reduced to 12.26 ± 5.78 dB, indicating a statistically significant change [16].

Primary stapes surgeries are more successful than revision surgeries [17], and hearing results are better after the first revision surgery than after multiple surgical procedures [18]. Revision surgeries in patients with otosclerosis require experienced surgeons [19, 20]. Lippy et al. retrospectively evaluated 522 revision stapedectomies over a 20-year period and reported that the best candidates for revision surgery are patients whose hearing improved after the previous surgery and then later decreased. The success of the revision surgery was reduced when the hearing remained the same or became worse after the previous surgery. Hearing deterioration is often associated with an increase in the number of revision procedures, otosclerosis regrowth especially should not be revised [21].

### PTA_4_ABG (N = 168)

Our study reports satisfactory audiological outcomes with the new MED-EL stapes prostheses. 65.5% and 91.7% of the patients reached a PTA_4_ ABG of ≤ 10 dB and ≤ 20 dB, respectively, and therefore were successfully rehabilitated. The mean PTA_4_ ABG of all patients was 9.1 ± 6.9 dB HL; only 14 patients had a post-operative PTA_4_ ABG of > 20 dB HL (range: 21.3–31.3 dB). A success rate of 64.8% and 61.0% was within 10 dB and a success rate of 94.3% and 100% was within 20 dB for one of the MED-EL stapes prostheses (mAXIS Stapes Prosthesis) reported by Beutner et al. [22] and Bevis et al. [23].

PTA_4_ ABG ≤ 10 dB and ≤ 20 dB were reported with comparable prostheses for 23.1–81.8% and 69.2–100% of the patients, respectively ([24–30]).

For the individual prostheses, 88.0–94.3% of the patients reached a PTA_4_ ABG of ≤ 20 dB; and 42.1–81.8% of the patients reached a PTA_4_ ABG of ≤ 10 dB (Table 2). When considering the PTA_4_ ABG of ≤ 20 dB, all prostheses have similar outcomes. The PTA_4_ ABG of ≤ 10 dB, mAXIS, mLOOP and mZAM Stapes Prostheses have better outcomes than the mFIX Stapes Prosthesis (42.1%). This PTA_4_ ABG of ≤ 10 dB outcome of the mFIX Stapes Prosthesis is comparable to other alternatives with a similar prosthesis design (38.6% [24] and 40.0% [28]). Additionally, it is evident that revision surgeries are less effective than initial implantations [17], which could also explain the different PTA_4_ ABG of ≤ 10 dB outcome of the mFIX Stapes prosthesis as compared to the other three prosthesis variants. However, due to the retrospective design of our study the information whether the studied PMEI implantation was an initial or revision surgery was not available.

According to the American Academy of Otolaryngology-Head and Neck Surgery the post-operative ABG should be monitored for at least one year [14]. Due to the retrospective design of our study, the follow-up times vary in every clinic. Since the follow-up is on average less than one year, the post-operative ABG is referred to as a technical hearing improvement rather than a clinical hearing improvement [14]. In our study, the post-operative follow-up time was less than one year, with the exception of the mFIX Stapes Prosthesis; however, the aim was not to show a hearing improvement, but percentage of patients achieving a post-operative PTA_4_ ABG of ≤ 20 dB.

### Crimping and audiological results

Multiple factors influence hearing outcomes. Tightness of the prosthesis fixation to the long process of the incus, crimping of the prosthesis head around the incus [31], pre-operative BC and ABG, type of surgery and the age of the patient all seem to predict the surgical outcome [26]. Patients who are older at the time of implantation (≥ 60 years of age) experience greater improvement than younger adults [32]. In stapes surgery, crimping is one of the most difficult steps and the outcomes are difficult to predict [30]. Optimal prosthesis fixation is essential for good sound transmission, but the process of manual crimping is risky (e.g., incus necrosis) for the middle and inner ear [31]. Therefore, crimping-free prostheses could be an advantage over crimping prostheses [24].

Handke et al. published a retrospective study with 190 patients implanted by the same surgeon. 112 patients were implanted with a Clip àWengen (crimp-free prosthesis) and 78 patients with a Matrix stapes prosthesis (crimp prosthesis) (Heinz Kurz GmbH, Dusslingen, Germany). It was shown that the ABG closure was significantly better at 0.5 kHz for the crimp prosthesis than for the crimp-free prosthesis [33].

In our study, when comparing the mean PTA_4_ ABG results of the crimp prostheses to the crimp-free prosthesis mFIX, the lowest PTA_4_ ABG was achieved at 2 kHz for both crimp mAXIS and mLOOP Stapes Prostheses, and at 3 kHz for the crimp mZAM Stapes Prosthesis **(**Table 4**)**.

**Table 4:** Comparison of the PTA_4_ ABG results between crimp-free and crimp prostheses

**Type of stapes prosthesis**	**Mean ± Standard Deviation of the post-operative PTA**_**4**_** ABG:**				
	**0.5 kHz** **[dB HL]**	**1 kHz** **[dB HL]**	**2 kHz** **[dB HL]**	**3 kHz** **[dB HL]**	**4 kHz** **[dB HL]**
mAXIS (crimping) (N = 88)	11.5 ± 9.5	9.3 ± 8.8	5.7 ± 7.4	8.7 ± 7.4	19.0 ± 11.2
mLOOP (crimping) (N = 50)	11.0 ± 11.5	10.2 ± 11.9	3.1 ± 8.4	8.8 ± 9.6	18.3 ± 14.9
mZAM (crimping) (N = 11)	10.0 ± 7.7	11.4 ± 8.4	6.8 ± 6.0	7.7 ± 7.7	10.0 ± 8.9
mFIX (crimp-free) (N = 19)	12.9 ± 5.3	11.2 ± 5.8	12.4 ± 8.4	13.9 ± 9.9	16.1 ± 7.6

### BC PTA_4_(N = 168)

Our study shows that the BC PTA_4_ was within the fluctuation range of ± 5 dB HL (test/retest error) for 159 (94.6%) of the 168 patients, with a mean PTA_4_ of 26.6 ± 10.3 dB HL pre-operatively and 23.9 ± 10.8 dB HL post-operatively.

In our study, 9 (5.4%) patients had a BC PTA_4_ deterioration of > 10 dB HL (range: 11.3–21.3 dB HL) when comparing pre- to post-operative BC PTA_4_ thresholds; 4 of these 9 patients were implanted with the mAXIS Stapes Prosthesis and 5 with the mLOOP Stapes Prosthesis. As our study does not separate between initial and revision stapes procedures, the 5.4% of patients with a BC PTA_4_ deterioration of > 10 dB HL is in the range of 4.7% –18%, reported by Schmid and Häusler [20], and Pedersen [34].

### Adverse events (N = 188)

Complaints usually occur in the first few weeks after the implantation and decrease with time [35].

12 (6.4%) of the 188 patients in our study reported a total of 13 AEs. For 3 AEs (in 3 patients), a relation to a device defect or surgical error could not be fully excluded, which should be discussed. Transient vertigo was reported in one (patient 163) of the patients (0.5%). 1 (patient 100) of the patients (0.5%) experienced hearing loss (BC deterioration of 18.8 dB HL), which was resolved with medication. 1 (patient 101) of the patients (0.5%) had a subjective feeling of hearing loss but showed no audiological hearing loss. No pre- and post-operative tinnitus was reported. The outcome of the stapes surgery depends on the surgical technique applied during the first surgery [19], the skill of the surgeon, and the characteristics of the prosthesis [36]. For perfect hearing reconstruction and consistently good hearing results, optimal prosthesis crimping is essential. Over- and under-crimping both have a negative effect on hearing results [37]. Over-crimping can lead to necrosis of the long process of the incus and under-crimping can lead to a post-operative residual ABG, or a reappearance of the CHL [28]. A stapedotomy technique that is too rigid, particularly in a patient with a thick stapes footplate, can lead to residual CHL. A prosthesis that is too short can result in poor hearing, while a prosthesis that is too long may cause vertigo and inner ear damage [38].

## Conclusion

Our study shows that stapes surgeries are associated with low prosthesis-related risks intra- and post-operatively. All four prosthesis versions are suitable candidates for reliable middle ear reconstruction, with similar results in the various groups in reaching the post-operative PTA_4_ ABG of ≤ 20 dB threshold.

In conclusion, MED-EL stapes prostheses present similar safety and audiological performance results to similar devices published elsewhere in the scientific literature. Nevertheless, the long-term audiological benefit and clinical safety remains to be demonstrated.

## Supplementary Information

Below is the link to the electronic supplementary material.

Supplementary file1 (DOCX 47 KB)
