# Supplementary material for: Clinical outcomes of 188 patients implanted with Med-El stapes prostheses
Source: Eur Arch Otorhinolaryngol. 2025 Oct 15;283(2):781–90. doi: 10.1007/s00405-025-09733-x (PMC12987915; doi:10.1007/s00405-025-09733-x)
Supplement: Supplementary file 1 — Supplementary file1 (DOCX 47 KB) [file 405_2025_9733_MOESM1_ESM.docx]

**ESM 1: Overview of patients:**

HL: hearing loss; MHL: mixed hearing loss; CHL: conductive hearing loss; NA: not applicable; post-op PTA_4_ ABG in bold: results is >20 dB; post-op BC PTA_4_ with a negative sign (-): improvement; post-op BC PTA_4_ with a positive sign (+): deterioration of >10 dB HL

| **mAXIS Stapes Prosthesis (N = 94)** | | | | | | | | | | | | | | | | |
| --- | --- | --- | --- | --- | --- | --- | --- | --- | --- | --- | --- | --- | --- | --- | --- | --- |
| **Patient ID** | | **Gender** | **Implanted ear** | | **Type of HL** | | **Age at surgery [years]** | | **Reason for PMEI implantation** | | **Etiology prior to the**  **PMEI implantation** | **Pre-op**  **BC PTA_4_**  **[dB HL]** | **Post-op**  **BC PTA_4_**  **[dB HL]** | **Post-op**  **AC PTA_4_**  **[dB HL]** | **Post-op PTA_4_ ABG**  **[dB]** | **Change in post-op**  **BC PTA_4_**  **[dB HL]** |
| 1 | | Female | Right | | MHL | | 34 | | Otosclerosis | | Not reported | 25.5 | 28.3 | 38.8 | 10.5 | +2.8 |
| 2 | | Male | Left | | MHL | | 35 | | Otosclerosis | | Not reported | 19.0 | 10.8 | 23.5 | 12.8 | -8.3 |
| 3* | | Female | Left | | MHL | | 39 | | Otosclerosis | | Not reported | NA | NA | NA | NA | NA |
| 4 | | Female | Left | | MHL | | 64 | | Otosclerosis | | Not reported | 33.5 | 29.0 | 39.3 | 10.3 | -4.5 |
| 5 | | Male | Right | | MHL | | 51 | | Otosclerosis | | Not reported | 18.5 | 32.8 | 36.3 | 3.5 | **+14.3** |
| 6 | | Male | Left | | CHL | | 41 | | Otosclerosis | | Not reported | 16.8 | 21.3 | 35.0 | 13.8 | +4.5 |
| 7 | | Female | Left | | MHL | | 51 | | Otosclerosis | | Not reported | 26.0 | 24.5 | 29.8 | 5.3 | -1.5 |
| 8 | | Male | Left | | MHL | | 44 | | Otosclerosis | | Not reported | 28.0 | 34.3 | 37.8 | 3.5 | +6.3 |
| 9 | | Female | Right | | MHL | | 49 | | Otosclerosis | | Not reported | 22.8 | 21.3 | 26.5 | 5.3 | -1.5 |
| 10 | | Female | Right | | MHL | | 48 | | Otosclerosis | | Not reported | 44.0 | 40.3 | 47.3 | 7.0 | -3.8 |
| 11 | | Female | Right | | MHL | | 34 | | Otosclerosis | | Not reported | 17.8 | 15.8 | 26.0 | 10.3 | -2.0 |
| 12 | | Female | Right | | CHL | | 41 | | Otosclerosis | | Not reported | 7.0 | 22.3 | 38.5 | 16.3 | **+15.3** |
| 13 | | Female | Left | | MHL | | 55 | | Otosclerosis | | Not reported | 27.5 | 30.0 | 38.8 | 8.8 | +2.5 |
| 14* | | Female | Right | | MHL | | 55 | | Otosclerosis | | Not reported | NA | NA | NA | NA | NA |
| 15 | | Female | Right | | MHL | | 39 | | Otosclerosis | | Not reported | 24.8 | 26.3 | 33.3 | 7.0 | +1.5 |
| 16 | | Female | Right | | CHL | | 70 | | Otosclerosis | | Not reported | 32.8 | 35.3 | 52.3 | 17.0 | +2.5 |
| 17 | | Male | Right | | CHL | | 63 | | Otosclerosis | | Not reported | 22.8 | 20.0 | 26.8 | 6.8 | -2.8 |
| 18 | | Male | Right | | CHL | | 42 | | Otosclerosis | | Not reported | 56.0 | 50.0 | 67.0 | 17.0 | -6.0 |
| 19 | | Female | Left | | MHL | | 67 | | Otosclerosis | | Not reported | 36.0 | 36.3 | 61.0 | **24.8** | +0.3 |
| 20 | | Female | Right | | CHL | | 44 | | Otosclerosis | | Not reported | 24.5 | 33.3 | 43.0 | 9.8 | +8.8 |
| 21 | | Female | Right | | CHL | | 54 | | Otosclerosis | | Not reported | 19.0 | 24.5 | 40.0 | 15.5 | +5.5 |
| 22 | | Male | Left | | MHL | | 46 | | Otosclerosis | | Not reported | 26.3 | 22.5 | 27.5 | 5.0 | -3.8 |
| 23* | | Female | Right | | MHL | | 76 | | Otosclerosis | | Not reported | NA | NA | NA | NA | NA |
| 24 | | Male | Left | | CHL | | 42 | | Otosclerosis | | Not reported | 17.5 | 17.5 | 21.3 | 3.8 | 0.0 |
| 25 | | Female | Left | | MHL | | 52 | | Otosclerosis | | Not reported | 42.5 | 37.5 | 38.8 | 1.3 | -5.0 |
| 26 | | Male | Left | | MHL | | 45 | | Otosclerosis | | Not reported | 51.3 | 52.5 | 57.5 | 5.0 | +1.3 |
| 27 | | Female | Left | | MHL | | 28 | | Otosclerosis | | Not reported | 42.5 | 33.8 | 37.5 | 3.8 | -8.8 |
| 28 | | Female | Left | | MHL | | 42 | | Otosclerosis | | Not reported | 21.3 | 25.0 | 27.5 | 2.5 | +3.8 |
| 29 | | Male | Left | | MHL | | 59 | | Otosclerosis | | Not reported | 32.5 | 27.5 | 37.5 | 10.0 | -5.0 |
| 30 | | Male | Right | | MHL | | 67 | | Otosclerosis | | Not reported | 28.8 | 32.5 | 35.0 | 2.5 | +3.8 |
| 31 | | Male | Left | | MHL | | 53 | | Otosclerosis | | Not reported | 28.8 | 26.3 | 26.3 | 0.0 | -2.5 |
| 32 | | Female | Left | | MHL | | 38 | | Otosclerosis | | Not reported | 27.5 | 23.8 | 23.8 | 0.0 | -3.8 |
| 33 | | Female | Left | | MHL | | 33 | | Otosclerosis | | Not reported | 36.3 | 32.5 | 43.8 | 11.3 | -3.8 |
| 34 | | Female | Left | | MHL | | 46 | | Traumatic  brain injury | | Traumatic brain injury | 38.8 | 32.5 | 36.3 | 3.8 | -6.3 |
| 35 | | Male | Left | | MHL | | 37 | | Otosclerosis | | Not reported | 21.3 | 21.3 | 25.0 | 3.8 | 0.0 |
| 36 | | Female | Right | | MHL | | 62 | | Otosclerosis | | Not reported | 31.3 | 31.3 | 31.3 | 0.0 | 0.0 |
| 37 | | Female | Right | | CHL | | 60 | | Otosclerosis | | Not reported | 12.5 | 25.0 | 36.3 | 11.3 | **+12.5** |
| 38 | | Male | Right | | CHL | | 64 | | Otosclerosis | | Not reported | 21.3 | 35.0 | 52.5 | 17.5 | **+13.8** |
| 39 | | Female | Left | | CHL | | 28 | | Revision  stapesplasty | | Otosclerosis | 11.3 | 8.8 | 11.3 | 2.5 | -2.5 |
| 40 | | Male | Right | | MHL | | 58 | | Otosclerosis | | Not reported | 35.0 | 31.3 | 38.8 | 7.5 | -3.8 |
| 41 | | Female | Left | | MHL | | 38 | | Otosclerosis | | Not reported | 18.8 | 23.8 | 33.8 | 10.0 | +5.0 |
| 42 | | Female | Left | | MHL | | 50 | | Otosclerosis | | Not reported | 30.0 | 37.5 | 42.5 | 5.0 | +7.5 |
| 43 | | Female | Left | | CHL | | 40 | | Otosclerosis | | Not reported | 26.3 | 21.3 | 31.3 | 10.0 | -5.0 |
| 44 | | Female | Right | | MHL | | 63 | | Otosclerosis | | Not reported | 37.5 | 38.8 | 52.5 | 13.8 | +1.3 |
| 45 | | Male | Right | | MHL | | 44 | | Otosclerosis | | Not reported | 26.3 | 25.0 | 48.8 | **23.8** | -1.3 |
| 46 | | Female | Left | | MHL | | 56 | | Revision  stapesplasty | | Otosclerosis | 22.5 | 23.8 | 26.3 | 2.5 | +1.3 |
| 47 | | Female | Right | | CHL | | 31 | | Otosclerosis | | Not reported | 15.0 | 12.5 | 35.0 | **22.5** | -2.5 |
| 48 | | Female | Right | | CHL | | 33 | | Otosclerosis | | Not reported | 17.5 | 8.8 | 17.5 | 8.8 | -8.8 |
| 49 | | Female | Left | | MHL | | 59 | | Otosclerosis | | Not reported | 43.8 | 41.3 | 51.3 | 10.0 | -2.5 |
| 50 | | Female | Right | | CHL | | 22 | | Otosclerosis | | Not reported | 23.8 | 21.3 | 26.3 | 5.0 | -2.5 |
| 51 | | Female | Left | | CHL | | 38 | | Otosclerosis | | Not reported | 11.3 | 12.5 | 21.3 | 8.8 | +1.3 |
| 52 | | Female | Left | | MHL | | 43 | | Revision  stapesplasty | | Otosclerosis | 20.0 | 17.5 | 28.8 | 11.3 | -2.5 |
| 53** | | Female | Right | | CHL | | 48 | | Otosclerosis | | Not reported | NA | NA | NA | NA | NA |
| 54 | | Female | Left | | CHL | | 26 | | Otosclerosis | | Not reported | 15.0 | 7.5 | 18.8 | 11.3 | -7.5 |
| 55 | | Female | Left | | MHL | | 55 | | Unknown | | Not reported | 32.5 | 31.3 | 40.0 | 8.8 | -1.3 |
| 56 | | Female | Left | | CHL | | 58 | | Otosclerosis | | Not reported | 31.3 | 26.3 | 35.0 | 8.8 | -5.0 |
| 57 | | Female | Left | | MHL | | 41 | | Otosclerosis | | Not reported | 26.3 | 20.0 | 32.5 | 12.5 | -6.3 |
| 58 | | Female | Right | | CHL | | 49 | | Revision  stapesplasty | | Otosclerosis | 21.3 | 16.3 | 22.5 | 6.3 | -5.0 |
| 59 | | Female | Right | | MHL | | 48 | | Revision  stapesplasty | | Otosclerosis | 18.8 | 13.8 | 22.5 | 8.8 | -5.0 |
| 60 | | Female | Left | | MHL | | 62 | | Otosclerosis | | Not reported | 36.3 | 38.8 | 46.3 | 7.5 | +2.5 |
| 61 | | Female | Left | | CHL | | 34 | | Otosclerosis | | Not reported | 20.0 | 8.8 | 11.3 | 2.5 | -11.3 |
| 62 | | Male | Left | | MHL | | 55 | | Unknown | | Not reported | 28.8 | 25.0 | 26.3 | 1.3 | -3.8 |
| 63 | | Female | Right | | CHL | | 29 | | Otosclerosis | | Not reported | 13.8 | 11.3 | 18.8 | 7.5 | -2.5 |
| 64 | | Female | Left | | MHL | | 36 | | Otosclerosis | | Not reported | 20.0 | 15.0 | 26.3 | 11.3 | -5.0 |
| 65 | | Female | Left | | MHL | | 61 | | Otosclerosis | | Not reported | 25.0 | 21.3 | 25.0 | 3.8 | -3.8 |
| 66 | | Male | Left | | MHL | | 44 | | Otosclerosis | | Not reported | 30.0 | 33.8 | 63.8 | **30.0** | +3.8 |
| 67 | | Male | Right | | CHL | | 50 | | Otosclerosis | | Not reported | 26.3 | 26.3 | 27.5 | 1.3 | 0.0 |
| 68 | | Female | Left | | CHL | | 36 | | Otosclerosis | | Not reported | 16.3 | 15.0 | 25.0 | 10.0 | -1.3 |
| 69 | | Female | Left | | CHL | | 24 | | Otosclerosis | | Not reported | 27.5 | 21.3 | 23.8 | 2.5 | -6.3 |
| 70 | | Female | Left | | CHL | | 42 | | Otosclerosis | | Not reported | 17.5 | 13.8 | 40.0 | **26.3** | -3.8 |
| 71 | | Female | Left | | CHL | | 21 | | Otosclerosis | | Not reported | 23.8 | 25.0 | 30.0 | 5.0 | +1.3 |
| 72 | | Female | Left | | CHL | | 59 | | Otosclerosis | | Not reported | 17.5 | 17.5 | 33.8 | 16.3 | 0.0 |
| 73 | | Male | Right | | MHL | | 51 | | Otosclerosis | | Not reported | 40.0 | 41.3 | 51.3 | 10.0 | +1.3 |
| 74 | | Male | Right | | MHL | | 50 | | Otosclerosis | | Not reported | 23.8 | 25.0 | 28.8 | 3.8 | +1.3 |
| 75 | | Male | Right | | CHL | | 38 | | Otosclerosis | | Not reported | 5.0 | 11.3 | 25.0 | 13.8 | +6.3 |
| 76 | | Female | Right | | MHL | | 34 | | Otosclerosis | | Not reported | 23.8 | 22.5 | 35.0 | 12.5 | -1.3 |
| 77 | | Female | Right | | MHL | | 67 | | Otosclerosis | | Not reported | 42.5 | 33.8 | 37.5 | 3.8 | -8.8 |
| 78 | | Female | Left | | CHL | | 26 | | Otosclerosis | | Not reported | 21.3 | 10.0 | 16.3 | 6.3 | -11.3 |
| 79 | | Male | Left | | CHL | | 64 | | Otosclerosis | | Not reported | 39.0 | 35.3 | 40.8 | 5.5 | -3.8 |
| 80 | | Male | Right | | MHL | | 42 | | Otosclerosis | | Not reported | 30.5 | 24.5 | 32.8 | 8.3 | -6.0 |
| 81 | | Female | Right | | CHL | | 56 | | Otosclerosis | | Not reported | 29.3 | 11.0*** | 15.0*** | 4.0*** | -16.5*** |
| 82 | | Female | Right | | CHL | | 41 | | Otosclerosis | | Not reported | 11.5 | 12.8 | 24.8 | 12.0 | +1.3 |
| 83 | | Female | Right | | CHL | | 44 | | Otosclerosis | | Revision | 27.0 | 14.5 | 27.3 | 12.8 | -12.5 |
| 84 | | Female | Left | | CHL | | 54 | | Otosclerosis | | Otosclerosis | 47.0 | 40.8 | 40.5 | -0.3 | -6.3 |
| 85 | | Female | Left | | MHL | | 28 | | Otosclerosis | | Not reported | 48.5 | 34.3 | 41.8 | 7.5 | -14.3 |
| 86 | | Female | Left | | MHL | | 77 | | Otosclerosis | | Not reported | 27.8 | 22.0 | 29.0 | 7.0 | -5.8 |
| 87 | | Female | Left | | CHL | | 35 | | Otosclerosis | | Otosclerosis | 21.0 | 19.8 | 22.8 | 3.0 | -1.3 |
| 88 | | Male | Right | | CHL | | 51 | | Otosclerosis | | No information | 15.3 | 12.5 | 23.8 | 11.3 | -2.8 |
| 89 | | Male | Right | | CHL | | 41 | | Otosclerosis | | Trauma in February 2021 | 30.5 | 23.3 | 35.5 | 12.3 | -7.3 |
| 90 | | Male | Left | | CHL | | 59 | | Otosclerosis | | Not reported | 24.8 | 8.8 | 21.0 | 12.3 | -16.0 |
| 91 | | Female | Right | | MHL | | 36 | | Otosclerosis | | Not reported | 49.3 | 37.8 | 47.8 | 10.0 | -11.5 |
| 92* | | Female | Right | | CHL | | 58 | | Otosclerosis | | Otosclerosis; alio loco surgeries | NA | NA | NA | NA | NA |
| 93* | | Male | Right | | CHL | | 58 | | Otosclerosis | | Not reported | NA | NA | NA | NA | NA |
| 94 | | Female | Right | | CHL | | 24 | | Otosclerosis | | Not reported | 13.5 | 4.0 | 14.5 | 10.5 | -9.5 |
| **mLOOP Stapes Prosthesis (N = 64)** | | | | | | | | | | | | | | | | |
| **Patient ID** | | **Gender** | **Implanted ear** | | **Type of HL** | | **Age at surgery [years]** | | **Reason for PMEI implantation** | | **Etiology** | **Pre-op**  **BC PTA_4_**  **[dB HL]** | **Post-op**  **BC PTA_4_**  **[dB HL]** | **Post-op**  **AC PTA_4_**  **[dB HL]** | **Post-op PTA_4_ ABG**  **[dB]** | **Change in post-op**  **BC PTA_4_**  **[dB HL]** |
| 95 | | Female | Right | | MHL | | 33 | | Otosclerosis | | Not reported | 30.0 | 26.3 | 52.5 | **26.3** | -3.8 |
| 96 | | Male | Right | | MHL | | 53 | | Otosclerosis | | Not reported | 22.5 | 18.8 | 27.5 | 8.8 | -3.8 |
| 97 | | Male | Left | | MHL | | 57 | | Otosclerosis | | Not reported | 25.0 | 17.5 | 26.3 | 8.8 | -7.5 |
| 98 | | Female | Left | | MHL | | 57 | | Otosclerosis | | Not reported | 30.0 | 28.8 | 52.5 | **23.8** | -1.3 |
| 99 | | Female | Right | | MHL | | 53 | | Otosclerosis | | Not reported | 22.5 | 23.8 | 25.0 | 1.3 | +1.3 |
| 100 | | Male | Right | | MHL | | 66 | | Otosclerosis | | Not reported | 20.0 | 33.8 | 41.3 | 7.5 | **+13.8** |
| 101 | | Female | Right | | CHL | | 50 | | Otosclerosis | | Not reported | 16.3 | 18.8 | 18.8 | 0.0 | +2.5 |
| 102 | | Female | Right | | MHL | | 34 | | Otosclerosis | | Not reported | 25.0 | 21.3 | 31.3 | 10.0 | -3.8 |
| 103* | | Female | Left | | MHL | | 61 | | Otosclerosis | | Not reported | NA | NA | NA | NA | NA |
| 104 | | Female | Left | | MHL | | 48 | | Otosclerosis | | Not reported | 33.8 | 30.0 | 35.0 | 5.0 | -3.8 |
| 105 | | Female | Left | | MHL | | 34 | | Otosclerosis;  Morbus Meniere  for 17 years | | Not reported | 20.0 | 10.0 | 13.8 | 3.8 | -10.0 |
| 106 | | Female | Left | | MHL | | 52 | | Otosclerosis | | Not reported | 55.0 | 50.0 | 56.3 | 6.3 | -5.0 |
| 107 | | Female | Left | | MHL | | 65 | | Otosclerosis;  revision stapesplasty | | Not reported | 23.8 | 26.3 | 35.0 | 8.8 | +2.5 |
| 108 | | Female | Left | | MHL | | 46 | | Chronic otitis media | | Not reported | 45.0 | 56.3 | 81.3 | **25.0** | **+11.3** |
| 109 | | Female | Right | | MHL | | 53 | | Otosclerosis in combination with tympanosclerosis | | Not reported | 30.0 | 21.3 | 42.5 | **21.3** | -8.8 |
| 110 | | Female | Left | | MHL | | 45 | | Otosclerosis | | Not reported | 27.5 | 20.0 | 20.0 | 0.0 | -7.5 |
| 111 | | Male | Left | | MHL | | 39 | | Otosclerosis | | Not reported | 38.8 | 21.3 | 21.3 | 0.0 | -17.5 |
| 112 | | Female | Left | | MHL | | 55 | | Otosclerosis with  an obliterating oval window | | Not reported | 28.8 | 26.3 | 30.0 | 3.8 | -2.5 |
| 113 | | Female | Left | | MHL | | 38 | | Otosclerosis | | Not reported | 22.5 | 18.8 | 21.3 | 2.5 | -3.8 |
| 114* | | Female | Right | | MHL | | 48 | | Otosclerosis | | Not reported | NA | NA | NA | NA | NA |
| 115 | | Male | Right | | MHL | | 51 | | Otosclerosis | | Not reported | 33.8 | 31.3 | 33.8 | 2.5 | -2.5 |
| 116* | | Male | Left | | MHL | | 52 | | Otosclerosis | | Not reported | NA | NA | NA | NA | NA |
| 117* | | Female | Left | | MHL | | 57 | | Otosclerosis | | Not reported | NA | NA | NA | NA | NA |
| 118 | | Female | Left | | MHL | | 65 | | Otosclerosis | | Not reported | 28.8 | 21.3 | 25.0 | 3.8 | -7.5 |
| 119 | | Female | Right | | MHL | | 50 | | Otosclerosis | | Not reported | 22.5 | 22.5 | 26.3 | 3.8 | 0.0 |
| 120 | | Male | Left | | MHL | | 57 | | Otosclerosis | | Not reported | 10.0 | 10.0 | 28.8 | 18.8 | 0.0 |
| 121 | | Female | Left | | MHL | | 48 | | Otosclerosis | | Not reported | 35.0 | 8.8 | 18.8 | 10.0 | -26.3 |
| 122 | | Female | Right | | MHL | | 39 | | Otosclerosis | | Not reported | 26.3 | 25.0 | 26.3 | 1.3 | -1.3 |
| 123 | | Female | Left | | MHL | | 60 | | Otosclerosis | | Not reported | 20.0 | 20.0 | 33.8 | 13.8 | 0.0 |
| 124* | | Female | Right | | MHL | | 60 | | Otosclerosis | | Not reported | NA | NA | NA | NA | NA |
| 125 | | Female | Right | | MHL | | 51 | | Otosclerosis | | Not reported | 21.3 | 7.5 | 21.3 | 13.8 | -13.8 |
| 126* | | Male | Right | | MHL | | 42 | | Otosclerosis | | Not reported | NA | NA | NA | NA | NA |
| 127 | | Female | Right | | MHL | | 61 | | Otosclerosis | | VSB with incus-SP coupler | 45.0 | 45.0 | 31.3 | -13.8 | 0.0 |
| 128* | | Male | Right | | MHL | | 64 | | Otosclerosis | | Not reported | NA | NA | NA | NA | NA |
| 129 | | Female | Right | | MHL | | 63 | | Otosclerosis | | Not reported | 37.5 | 22.5 | 23.8 | 1.3 | -15.0 |
| 130* | | Female | Left | | MHL | | 64 | | Otosclerosis | | Not reported | NA | NA | NA | NA | NA |
| 131 | | Female | Left | | MHL | | 47 | | Otosclerosis | | Not reported | 21.3 | 7.5 | 22.5 | 15.0 | -13.8 |
| 132* | | Female | Left | | MHL | | 26 | | Otosclerosis | | Not reported | NA | NA | NA | NA | NA |
| 133 | | Male | Right | | MHL | | 57 | | Otosclerosis | | Not reported | 31.3 | 25.0 | 35.0 | 10.0 | -6.3 |
| 134* | | Female | Right | | MHL | | 36 | | Otosclerosis | | Not reported | NA | NA | NA | NA | NA |
| 135 | | Female | Right | | MHL | | 24 | | Otosclerosis | | Not reported | 37.5 | 31.3 | 32.5 | 1.3 | -6.3 |
| 136 | | Male | Right | | MHL | | 51 | | Otosclerosis | | Not reported | 23.8 | 22.5 | 22.5 | 0.0 | -1.3 |
| 137 | | Male | Left | | MHL | | 45 | | Otosclerosis | | Not reported | 25.0 | 20.0 | 28.8 | 8.8 | -5.0 |
| 138 | | Male | Right | | MHL | | 65 | | Otosclerosis | | Not reported | 37.5 | 30.0 | 38.8 | 8.8 | -7.5 |
| 139 | | Female | Right | | MHL | | 36 | | Otosclerosis | | Not reported | 25.0 | 20.0 | 22.5 | 2.5 | -5.0 |
| 140* | | Male | Right | | MHL | | 74 | | Otosclerosis | | Not reported | NA | NA | NA | NA | NA |
| 141 | | Female | Left | | MHL | | 49 | | Otosclerosis | | Not reported | 13.8 | 27.5 | 32.5 | 5.0 | **+13.8** |
| 142 | | Female | Right | | MHL | | 55 | | Otosclerosis | | Not reported | 25.0 | 36.3 | 37.5 | 1.3 | **+11.3** |
| 143 | | Female | Right | | MHL | | 68 | | Otosclerosis | | Not reported | 50.0 | 50.0 | 61.3 | 11.3 | 0.0 |
| 144* | | Female | Left | | MHL | | 59 | | Otosclerosis | | Not reported | NA | NA | NA | NA | NA |
| 145 | | Female | Right | | CHL | | 12 | | Otosclerosis | | Not reported | 10.0 | 7.5 | 30.0 | **22.5** | -2.5 |
| 146 | | Female | Left | | MHL | | 41 | | Otosclerosis | | Not reported | 40.0 | 26.3 | 28.8 | 2.5 | -13.8 |
| 147 | | Female | Right | | MHL | | 46 | | Otosclerosis | | Not reported | 18.8 | 11.3 | 11.3 | 0.0 | -7.5 |
| 148 | | Male | Right | | MHL | | 47 | | Otosclerosis | | Not reported | 31.3 | 52.5 | 68.8 | 16.3 | **+21.3** |
| 149 | | Male | Right | | MHL | | 18 | | Otosclerosis | | Not reported | 32.5 | 2.5 | 11.3 | 8.8 | -30.0 |
| 150 | | Female | Left | | MHL | | 35 | | Otosclerosis | | Not reported | 27.5 | 37.5 | 46.3 | 8.8 | +10.0 |
| 151* | | Male | Left | | CHL | | 46 | | Otosclerosis | | Not reported | NA | NA | NA | NA | NA |
| 152 | | Female | Right | | MHL | | 47 | | Otosclerosis | | Not reported | 20.0 | 10.0 | 18.8 | 8.8 | -10.0 |
| 153 | | Female | Left | | MHL | | 53 | | Otosclerosis | | Not reported | 12.5 | 22.5 | 37.5 | 15.0 | +10.0 |
| 154* | | Male | Right | | MHL | | 44 | | Otosclerosis | | Not reported | NA | NA | NA | NA | NA |
| 155 | | Female | Left | | MHL | | 39 | | Otosclerosis | | Not reported | 26.3 | 15.0 | 20.0 | 5.0 | -11.3 |
| 156 | | Female | Left | | MHL | | 59 | | Otosclerosis | | Not reported | 43.8 | 37.5 | 40.0 | 2.5 | -6.3 |
| 157 | | Female | Right | | MHL | | 40 | | Otosclerosis | | Not reported | 22.5 | 18.8 | 30.0 | 11.3 | -3.8 |
| 158 | | Female | Right | | CHL | | 57 | | Otosclerosis | | Not reported | 12.5 | 17.5 | 48.8 | **31.3** | +5.0 |
| **mZAM Stapes Prosthesis (N = 11)** | | | | | | | | | | | | | | | | |
| **Patient ID** | | **Gender** | **Implanted ear** | | **Type of HL** | | **Age at surgery [years]** | | **Reason for PMEI implantation** | | **Etiology** | **Pre-op**  **BC PTA_4_**  **[dB HL]** | **Post-op**  **BC PTA_4_**  **[dB HL]** | **Post-op**  **AC PTA_4_**  **[dB HL]** | **Post-op PTA_4_ ABG**  **[dB]** | **Change in post-op**  **BC PTA_4_**  **[dB HL]** |
| 159 | | Female | Left | | MHL | | 51 | | Otosclerosis | | No | 38.8 | 30.0 | 41.3 | 11.3 | 8.8 |
| 160 | | Female | Right | | MHL | | 46 | | Otosclerosis | | Not reported | 20.6 | 18.1 | 26.3 | 8.1 | 2.5 |
| 161 | | Male | Left | | CHL | | 53 | | Otosclerosis | | Not reported | 11.3 | 12.5 | 20.6 | 8.1 | +1.3 |
| 162 | | Female | Right | | CHL | | 14 | | Congenital ear  defect | | Not reported | 15.0 | 15.6 | 21.9 | 6.3 | -0.6 |
| 163 | | Female | Left | | MHL | | 36 | | Otosclerosis | | Not reported | 23.1 | 20.6 | 30.6 | 10.0 | 2.5 |
| 164 | | Male | Right | | CHL | | 40 | | Otosclerosis | | Not reported | 12.5 | 18.1 | 25.0 | 6.9 | +5.6 |
| 165 | | Male | Right | | MHL | | 46 | | Otosclerosis | | Not reported | 27.5 | 31.3 | 59.4 | **28.1** | +3.8 |
| 166 | | Male | Right | | MHL | | 45 | | Otosclerosis | | Not reported | 21.3 | 13.1 | 19.4 | 6.3 | 8.1 |
| 167 | | Male | Right | | MHL | | 60 | | Otosclerosis | | Not reported | 18.8 | 16.9 | 24.4 | 7.5 | 1.9 |
| 168 | | Female | Left | | MHL | | 56 | | Otosclerosis | | No | 32.5 | 30.0 | 31.3 | 1.3 | 2.5 |
| 169 | | Female | Left | | MHL | | 35 | | Otosclerosis | | No | 12.5 | 17.5 | 22.5 | 5.0 | +5.0 |
| **mFIX Stapes Prosthesis (N = 20)** | | | | | | | | | | | | | | | | |
| **Patient ID** | **Gender** | | | **Implanted ear** | | **Type of HL** | | **Age at surgery [years]** | | **Reason for PMEI implantation** | **Etiology** | **Pre-op**  **BC PTA_4_**  **[dB HL]** | **Post-op**  **BC PTA_4_**  **[dB HL]** | **Post-op**  **AC PTA_4_**  **[dB HL]** | **Post-op PTA_4_ ABG**  **[dB]** | **Change in post-op**  **BC PTA_4_**  **[dB HL]** |
| 170 | Female | | | Left | | MHL | | 39 | | Otosclerosis | Not reported | 23.0 | 13.0 | 22.0 | 9.0 | -10.0 |
| 171 | Male | | | Right | | MHL | | 35 | | Otosclerosis | Not reported | 13.5 | 10.0 | 24.5 | 14.5 | -3.5 |
| 172 | Female | | | Left | | MHL | | 30 | | Otosclerosis | Not reported | 18.0 | 5.8 | 18.0 | 12.3 | -12.3 |
| 173 | Male | | | Right | | CHL | | 25 | | Head trauma | Not reported | 12.5 | 10.0 | 21.3 | 11.3 | -2.5 |
| 174 | Female | | | Right | | CHL | | 26 | | Otosclerosis | Not reported | 25.0 | 26.3 | 35.0 | 8.8 | +1.3 |
| 175 | Female | | | Left | | MHL | | 69 | | Otosclerosis; gold piston dislocation; incus necrosis | Prior alio loco surgeries | 33.8 | 20.0 | 30.0 | 10.0 | -13.8 |
| 176* | Male | | | Right | | CHL | | 26 | | Otosclerosis | Not reported | NA | NA | NA | NA | NA |
| 177 | Female | | | Left | | MHL | | 40 | | Otosclerosis | Not reported | 35.0 | 31.3 | 36.3 | 5.0 | -3.8 |
| 178 | Female | | | Right | | CHL | | 39 | | Otosclerosis | Not reported | 18.8 | 20.0 | 30.0 | 10.0 | +1.3 |
| 179 | Female | | | Right | | CHL | | 25 | | Otosclerosis | Not reported | 15.0 | 10.0 | 30.0 | 20.0 | -5.0 |
| 180 | Male | | | Right | | MHL | | 32 | | Otosclerosis | Not reported | 26.3 | 16.3 | 30.0 | 13.8 | -10.0 |
| 181 | Male | | | Left | | MHL | | 67 | | Otosclerosis | Not reported | 43.8 | 37.5 | 60.0 | **22.5** | -6.3 |
| 182 | Female | | | Right | | MHL | | 43 | | Otosclerosis | Not reported | 48.8 | 33.8 | 51.3 | 17.5 | -15.0 |
| 183 | Male | | | Right | | MHL | | 82 | | Otosclerosis | Not reported | 52.5 | 52.5 | 75.0 | **22.5** | 0.0 |
| 184 | Female | | | Right | | CHL | | 44 | | Otosclerosis | Not reported | 23.8 | 15.0 | 21.3 | 6.3 | -8.8 |
| 185 | Male | | | Right | | CHL | | 53 | | Otosclerosis | Not reported | 16.3 | 16.3 | 25.0 | 8.8 | 0.0 |
| 186 | Male | | | Right | | MHL | | 57 | | Otosclerosis | Not reported | 28.8 | 25.0 | 37.5 | 12.5 | -3.8 |
| 187 | Male | | | Right | | CHL | | 28 | | Cranial trauma | Not reported | 17.5 | 13.8 | 15.0 | 1.3 | -3.8 |
| 188 | Male | | | Right | | MHL | | 54 | | Otosclerosis | Not reported | 30.0 | 27.5 | 41.3 | 13.8 | -2.5 |
| 189 | Female | | | Right | | MHL | | 57 | | Accident | Not reported | 34.8 | 22.5 | 42.5 | 20.0 | -12.3 |

*N=20 patients (mAXIS: patient 3, 14, 23, 92, 93; mLOOP: patient 103, 114, 116, 117, 124, 126, 128, 130, 132, 134, 140, 144, 151, 154; mZAM: NA; mFIX: patient 176): were analyzed for AEs only, but not for audiological results

******N=1 (patient 53): was excluded from the study, because of missing follow-up information

***N=1 (patient 81): PTA_4_ was calculated with 4 kHz, instead of 3 kHz (3 kHz was missing)
